# Functionalized graphene/polystyrene composite, green synthesis and characterization

**DOI:** 10.1038/s41598-022-26270-3

**Published:** 2022-12-16

**Authors:** Rania Farouq

**Affiliations:** Petrochemical Engineering Department, Pharos University in Alexandria, Alexandria, Egypt

**Keywords:** Materials for energy and catalysis, Chemical engineering

## Abstract

A composite of sulfonated waste polystyrene (SWPS) and graphene oxide was synthetized by an inverse coprecipitation in-situ compound technology. Polystyrene (PS) has a wide range of applications due to its high mechanical property. the graphene were incorporated into sulfonated polystyrene (SPS) to improve the thermal stability and mechanical performance of the composites. Functionalized graphene were synthesized with tour method by using recovered anode (graphite) of dry batteries while sulfonated waste expanded polystyrene was obtained through sulfonation of the polymer. The SPS and GO + SPS composite were characterized using by Fourier Transform Infrared spectroscopy (FT-IR) and transmission electron microscopy (TEM). While the degree of sulfonation (DS) was determined through elemental analysis. The results show the degree of sulfonation of the composite is 23.5% and its ion exchange capacity is 1.2 meq g^−1^. TEM analysis revealed that the GO particles were loaded on the surface of sulphonated polystyrene and that the SWPS was intercalated into the sub-layers of nanoG homogeneously, which result in an increase in electrical conduction.

## Introduction

With up to millions tonnes of plastic released up to now, plastic garbage is becoming a significant environmental concern. Of these waste streams, one of the largest is that of polystyrene (PS), with an annual production rate exceeding 20 million tons per year^1^, waste polystyrene can be chemically modified through various processes like sulfonation^2,3^. Hydrophilicity, proton conductivity, ion exchange capacity, and water solubility are improves when polymers are sulfonated^4–7^. Biocompatible electrodes, stimuli-responsive photonic crystals, humidity sensors, parts of photovoltaic systems, ion exchange membranes, flocculant compound for water treatment, and catalysts are just a few of the uses for sulfonated waste polystyrene^3,8^.

Dry cell batteries are extensively employed in different of home applications. These batteries cannot be recharged and must be disposed after they have been totally consumed. Huge amounts of spent batteries wastes are produced, which need to be recycled to preserve raw materials in the interest of the sustainable development^9,10^. The most of battery recycling technologies focus only on recovering precious metals such as Zn, Mn, Fe, and other metals. The graphite in the batteries is burned, left as residue, or ignored throughout the manufacturing process. Anyone considering recycling or reusing these batteries should consider the graphite rod located in the battery's core^11^. Graphene is drawing attention as a catalyst support because of its stability and compatibility with a wide range of catalytically active particles^12^. Negatively charged groups on a functionalized graphene surface such as graphene oxide (GO), interfere with dyes,metallic ions, and organic compounds^13^. The high conductivity of graphene oxide can aid electron transport during transformations^14^.

The interaction between graphene and the supported metal catalyst is usually weak due to the chemically inert nature of graphene, and thus defects and functional groups are instilled to improve this interaction^15^.

GO improve the thermal, electrically conductive, electrorheological and adsorptive properties of polymer composites (Hierarchical assembly of polystyrene/graphitic carbon nitride/reduced graphene oxide nanocomposites toward high fire safety). graphene oxide (GO) nanosheets used in memebrane matrix demonstrated positive effects on the performance of fuel cells^16^. Its large surface area with many polarized groups may help to construct continuous proton transport channels and resist the transportation of methanol, so a small amount of GO could be beneficial for improving the proton conducting behaviors and lower the methanol permeability^17^.

The most widely used Hummers’method for synthesis of GO involved treatment of graphite with KMnO_4_ and NaNO_3_ which is considered to be hazardous. When compared to Hummers' technique, Tour employs H_3_PO_4_, a weak acid with minimal toxicity and no risk of gas release. It is also regarded to be more sustainable^18^. This technique produces a graphite oxide that is more oxidized with more regular carbon skeleton^19–21^. In this work, we first utilized an environmentally friendly route to prepare a graphene derivate (graphene oxide, GO). The GO was then incorporated into the SWPS particles which can be used as ion exchange. Although ion exchange is one of the most efficient methods for heavy metal removal, with technologically simple operation, high efficiency and reliability. However, due to the high cost of ion exchange resins, the ion exchange process is not commonly used for industrial wastewater treatment. So using recycled materials as ion exchange resins are also a possible solution for cost reduction production and application of ion exchange resins The composite of sulfonated waste polystyrene and graphene oxide nanoparticles was synthesized and characterized.

## Materials and methods

### Extraction of graphite from waste dry cell batteries

The spent batteries were carefully disassembled without causing any damage to the graphite rods within. To remove the other chemicals adhered to the graphite rods, they were disassembled and thoroughly wiped and washed with distilled water. After drying, the electrodes were ground and crushed to produce a fine graphite powder. Further treatment of the graphite powder was carried out in a beaker, containing HCl and HNO_3_ (3:1) and heated for 6 h. Finally, to get the pH back to normal, the sample was centrifuged and rinsed multiple times in distilled water. Recovered graphite powder [G (R)] was dried at 60 °C for 24 h.

### Preparation of graphene oxide by a modified hummer’s method

Graphene oxide (GO) was synthitized from graphite powder using a modified hummers technique in a conventional approach. Sulfuric acid (H_2_SO_4_) and phosphoric acid (H_3_PO_4_) (ratio 9:1) were blended for few minutes. Then, 3.75 g of graphite was added to the reaction mixture while stirring. After that, 22.5 g of potassium permanganate (KMnO_4_) was gently added to the mixture. The solution was agitated for 24 h until it turned dark green. 30 ml hydrogen peroxide (H_2_O_2_) was dropped gently and agitated for 10 min to remove excess KMnO4. The reaction is exothermic so it was allowed to cool down. 400 ml of equal voulms of hydrochloric acid (HCl) and deionized water mixture was added and centrifuged for 20 min at 5000 rpm. The solution was then decanted away, and the residuals were rewashed three times with HCl and water. Then the solution was dried at 90 °C for 24 h.

### Preparation of sulfonated polystyrene (SPS)

Waste EPS packaging material was collected washed, and air dried. It was ground in a seed mill until the pieces were from 3 to 7 mm wide with a thickness of 1 mm. The resulting polystyrene was sulfonated in two stages by reaction with acetyl sulfate in CH_2_Cl_2_, according to the following sulfonation process:

#### Preparation of acetyl sulphate (sulfonation reagent)

This step should be done freshly prior to the sulfonation reaction of PS. 12 mL of acetic acid anhydride were mixed with 24 mL of dichloroethane, in three-neck flask under nitrogen atmosphere. This solution is cooled to 0 °C in ice bath. Then, 6 mL of Oleum were added portion-wise and stirred for 1 h while maintaining the temperature, resulting in 1 M of acetyl sulphate (homogeneous and clear solution).

Note: during the preparation an excess of acetic anhydride were used to make sure that there are no water traces, and that the latter was completely converted to [acetyl sulphate]. Finally, the three-neck flask was capped, and the acetyl sulphate resulted in DCE solution was ready to be used.

#### Sulfonation reaction

4 g of PS were completely dissolved with 200 mL of dichloroethane in a 500 mL three-neck flask, equipped with a condenser, mechanical stirring, thermometer and dropping funnel. The solution was stirred at 40 °C under a small nitrogen flow rate, until a homogenous solution is attained. As prepared 1 M acetyl sulphate was immediately transferred and added to the polymer solution at (40 °C) using a dropping funnel. The degree of sulfonation of PS was controlled by adjusting the sulfonation time from 1 to 6 h, the solution became clear yellow colour after the addition of sulfonating agent. The reaction was stopped by adding 10 mL of isopropanol to the mixture and allowing it to cool to room temperature. Next step was precipitation by dropping the prepared solution into a large volume of boiling deionized water. Followed by washing several times with deionized water to eliminate the solvent and hydrolyse the acetyl sulphate. Finally, the obtained powder was filtered and dried at (70 °C) in a vacuum oven for 3 days.

### Synthesis of GO + SWPS composites

**Figure 1:**
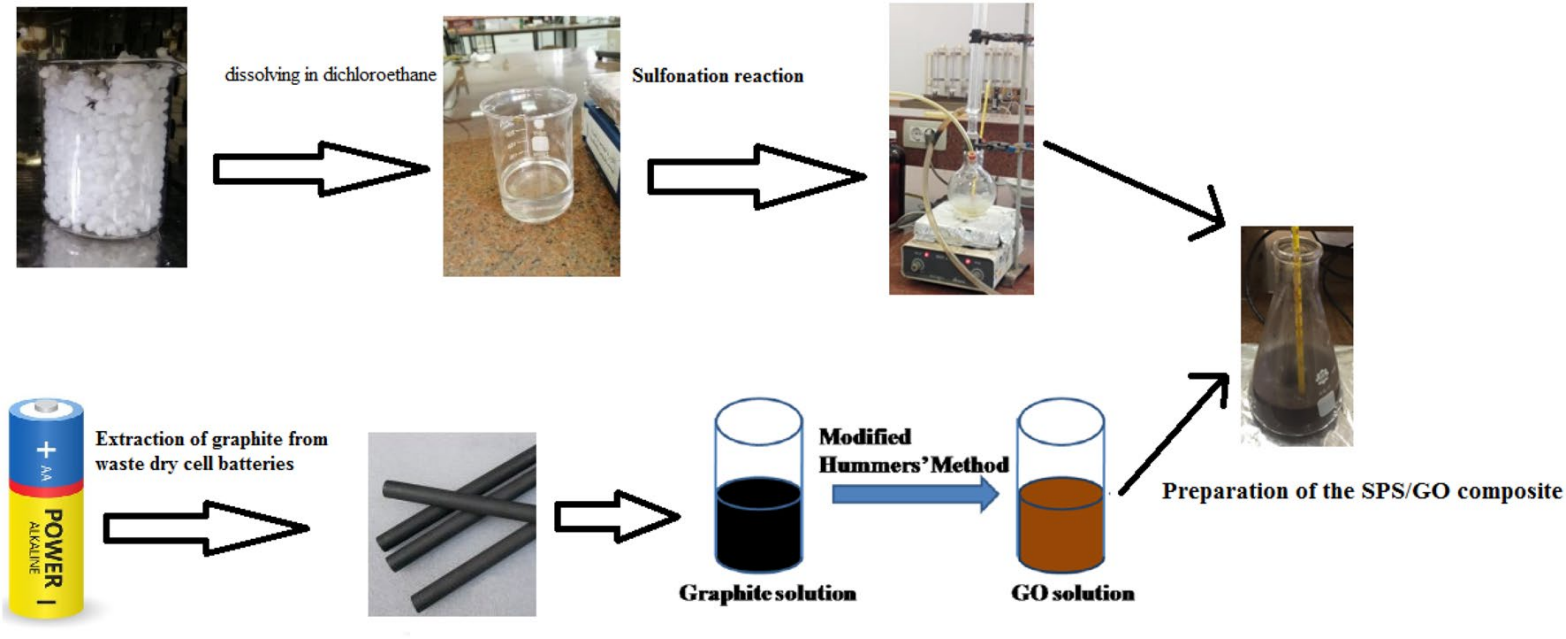
Synthesis of GO + SWPS composites.

Inverse coprecipitation in-situ compound method was used to form SPS/GO composites as shown in Fig. 1. 2 g SPS particles and 40 mL of 0.2 M NaOH solution were transferred to a 250 mL four-neck flask and kept at 80 °C for 30 min. Throughout the reaction, N_2_ was bubbled. Then, using an ultrasonic disperse technique, 0.0164 g GO were dispersed into 60 mL 1:1 (V/V) ethanol–water mixed solvents. After that, the mixture was dropped into the above four-neck flask and vigorously stirred for 30 min at 80 °C. The suspension's colour changed to black almost instantly. When the dropping was done, the stirring was maintained at 50 °C for 1 h. The composite was isolated by centrifuge after cooling to room temperature, rinsed with deionized water until the solution was neutral, then dried under vacuum at 60 °C for 24 h^21^.

## Results & discussion

### Reaction scheme of homogeneous sulfonation

**Figure 2:**
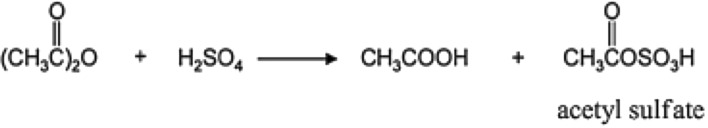
the reactions involved in the homogeneous sulfonation of PS.

**Figure 3:**
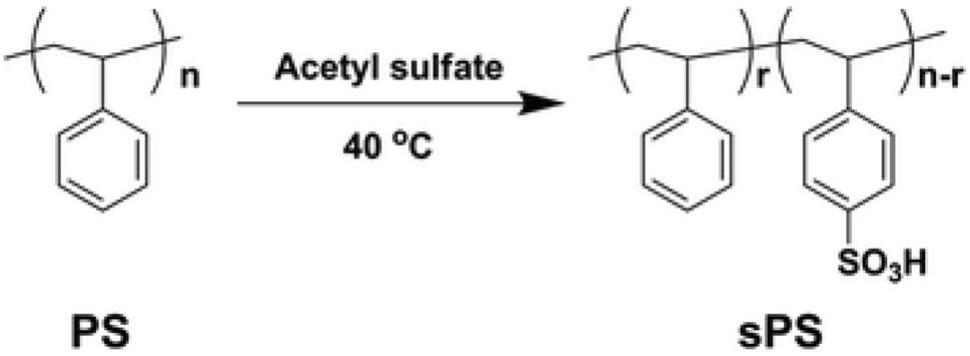
Reaction scheme of homogeneous sulfonation.

The sulphonation process occurs in two steps: Fig. 2 depicts the reaction involved in homogeneous sulfonation of PS, and Fig. 3 depicts the homogeneous sulfonation reaction scheme.

**Figure 4:**
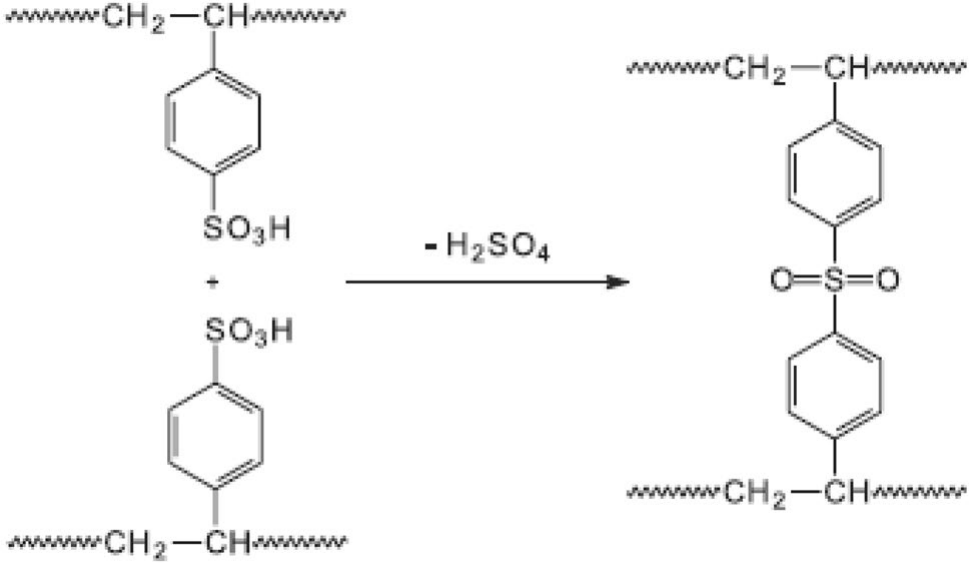
Crosslinking reaction of two sps groups.

Sulfonation reaction might take longer than expected because of a side reaction for sulfonate synthesis induced by a crosslinking event that occurred between two sulfonic groups for distinct SPS units due to an intermolecular mechanism. Figure 4 depicts the crosslinking mechanism.

Tendency to the inter-molecular reaction is affected with several parameters including:

1. The sulfonic groups content will be increased by the elevating the concentration for the reaction sulfonating agent used with polymer solution.
2. Also elevating reaction temperature will lead to a higher percentage of the yields

The best degree of sulfonation 30% was found to be achieved after 3 h^22^.

### FT-IR characterization

**Figure 5:**
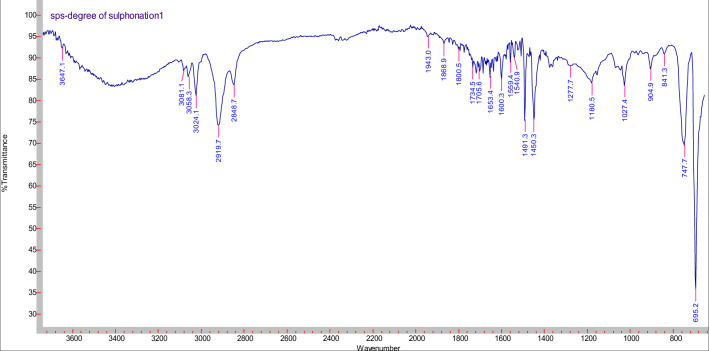
FTIR spectra of the sulfonated polystyrene.

There are principle peaks found in sulfonated polystyrene represented in Fig. 5, the band characteristic of the aromatic =C–H stretching band at 3024.1 cm^−1^, the band characteristic of the symmetrical stretch of CH_2_ is also observed in 2848.7 cm^−1^ –CH_2_—asymmetric stretching sharp peak a 2919.7 cm^−1^, C=C para—disubstituted benzene at 1450.3 cm^−1^, The vibration of the aromatic skeleton is found in the band at 1600 cm^−1^, The bands between 904.9 and 695.2 cm^−1^ are associated with vibration of C–H deformation of the aromatic ring which are characteristic to polystyrene .

The peak at 3647.1 cm^−1^ belongs to O–H in the sulfonic acid groups, stretching vibration of S=O at 1180.5 cm^−1^, and symmetric stretching of the O=S=O at (1027.4) indicates qualitatively the presence of the attached –SO_3_H groups in each of sulfonated samples. The band related to the substituted benzene in the para position (841.3 cm^−1^) validates the aromatic ring substitution.

**Figure 6:**
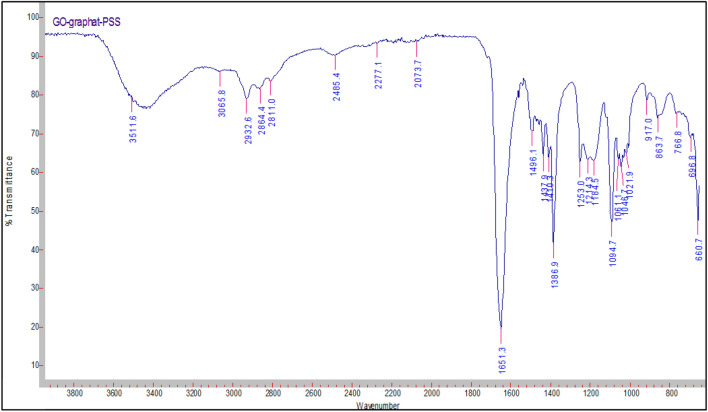
FTIR spectra of the SPS/GO composite.

Because both SPS and GO contain a large number of functional groups on their surfaces, it's possible to predict significant interactions between the two to cause composite production. Actually, as evidenced by the IR data described below, this is proven to be accurate. Figure 6 depicts the key stretching frequencies of SPS/GO. Both SPS and GO peaks are observed in the composite, suggesting that both components are present. The O–H stretching of –SO_3_H of SPS at 3647.1 cm^−1^ shifted to lower wavenumber (3511.6 cm^−1^) and becomes broader in case of composite attributing that –SO_3_H of SPS is involved in the hydrogen bonding interactions with GO particles. Similarly the shifting of asymmetric stretching of –SO_3_H of SPS from 1180.5 to 1094.7 cm^−1^ in composite indicates the presence of hydrogen bonding interactions between the SPS and GO. Also, a substantial shift (695.2–660.7 cm^−1^) of out of plane blending vibration of PS ring in case of composite is the indication of *π*–*π* interactions between the PS backbone and basal planes of graphene. These findings clearly show significant interactions between SPS and GO, which resulted in the formation of a stable composite. The peak of GO is due to C–OH stretching is observed at wavenumber at 1253 cm^−1^

### Structural studies of SPS/GO composites: TEM analysis

TEM were used to analyze the structure of the as-prepared composite. The sample was dissolved in little amount of DMF and the solution is diluted with water and then the TEM samples were prepared by drop casting samples on the carbon coated copper (200 mesh) grid.

**Figure 7:**
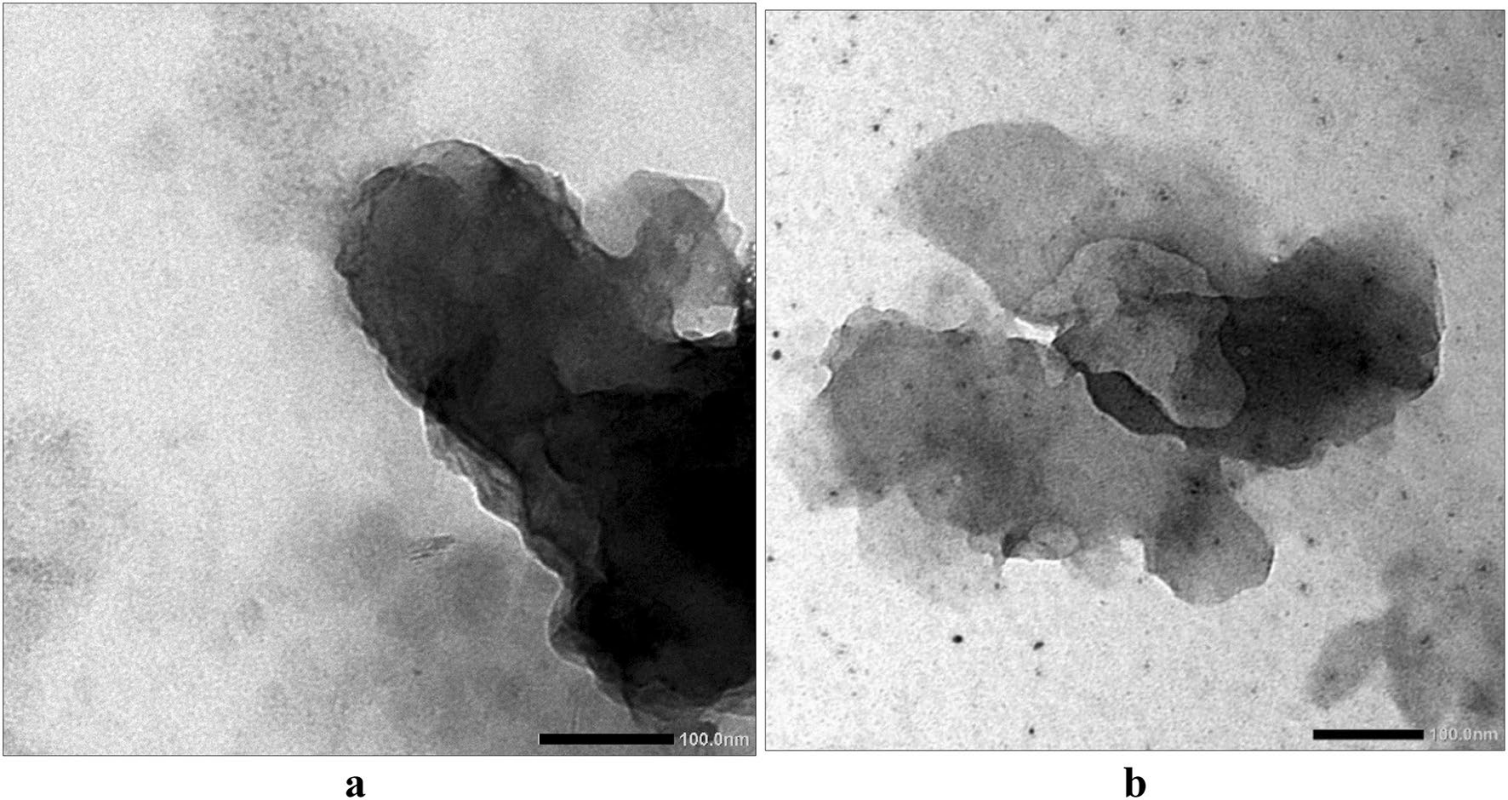
TEM analysis depicting the distribution of (**a**) graphene oxide (GO) (**b**) GO/SPS.

These results are in accordance with the TEM image of SPS/GO composite observation. In Fig. 7a, it can be seen that particles were loaded on the surface of sulphonated polystyrene exhibiting a homogeneous distribution with no aggregation compared to Fig. 7b of the GO which shows the linear deposit, exhibiting clear coagulation. The GO-SPS show the few-layered graphene oxide grafted with branches of SPS. This indicates that SPS particles are effectivlly coated by GO particles.

### The degree of sulfonation (DoS)

The resulting SPS was determined by titration. A predetermined amount of SPS were dispersed into 50 mL of distilled water with ultrasonication and viguros agitation for 30 min. Two droplets of phenolphthalein solution were added. The mixture was titrated by NaOH solution. The consumed volume of NaOH solution was recorded. The DoS was calculated by following Eq.:

1 $$ DoS = V_{NaOH } *C_{NaOH } /M $$

where C_NaOH_ is the concentration of NaOH and M is the mass of SPS.

As the number of sulfonic acid groups linked to the PS has increased, the SPS is more soluble in water and better able to interact with the graphene. As a result of the high degree of substitution of PS by sulfonic acid groups, the phenyl groups can exhibit a conjugation effect with sulfonic acid groups, confirmed by the increased electrical conductivity of the SWPS matrix. As the sulfonation increases, and the sulfonic groups interconnect result in the creation of ionic nanochannels. Those ionic nanochannels are critical for the transport and mechanical properties of the polymer^23^.

DS depends largely on many factors such as the concentration of polymer, concentration of the sulfonating agent, the temperature and the time of sulfonation reaction. Indeed, the degree of sulfonation could be controlled by adjusting these parameters. It was clear that, as the sulfonation reaction time was increased, a noticeable increase in DS^24^.

## Conclusions

A composite of graphene oxide (GO) and sulfonated polystyrene are prepared with enhanced properties. The manufacture of SPS was observed in the Fourier transform infrared spectra FT-IR as created function group peak vibration range. The properties of SPS are favourably manipulated by the incorporation of GO. Intermolecular interactions between the components in composite are established by FTIR. Composite is characterized by transmission electron microscopy (TEM) which showed the uniform distribution of GO particles in SPS matrix.

Because of the strong interaction between –SO_3_H functionality of SPS and functional group of GO, the SPS particles are adsorbed on the graphene surface. This results in stable SPS/GO composite. The composite display better thermal, mechanical and electrical conduction compared to polymer.

SPS particles help to produce and stabilize single layer graphene sheets; in turn these GO Sheets help to produce mechanically strong ion conducting SPS resin. This enhancement allows us to use it for waste water treatment as durable and efficient raw material able to stand in severs conditions. The method we proposed in this paper is a high standard method with several advantages, including the use of waste polystyrene, which reduces environmental pollution, avoids harmful gases, which are environmentally beneficial, and is a convenient and straightforward procedure that saves energy resources. We hope for more practical time leading us for more detail and results but COVID-19 waste mostly available time to finish this study.

## Data Availability

The datasets used and/or analyzed during the current study available from the corresponding author on reasonable request.
